# Survey-Based Analysis of Current Trends for Prescribing Gastrointestinal Protectants among Small-Animal General Practitioners in Portugal

**DOI:** 10.3390/vetsci8050070

**Published:** 2021-04-23

**Authors:** Rita Baptista, Ryane Englar, Berta São Braz, Rodolfo Oliveira Leal

**Affiliations:** 1 Hospital Escolar Veterinário, Faculdade de Medicina Veterinária—Universidade de Lisboa, Av. Universidade Técnica, 1300-477 Lisbon, Portugal; rita.baptista@hvetoeste.com (R.B.); bsaobraz@fmv.ulisboa.pt (B.S.B.); 2 Hospital Veterinário do Oeste, EN 247 Condomínio Valpraia Loja B, Vale de Adares, 2530-086 Lourinhã, Portugal; 3 College of Veterinary Medicine, University of Arizona, 1580 E Hanley Blvd., Oro Valley, AZ 85737, USA; renglar@arizona.edu; 4 CIISA-Centro de Investigação Interdisciplinar em Sanidade Animal, Faculdade de Medicina Veterinária, Universidade de Lisboa, Av. Universidade Técnica, 1300-477 Lisbon, Portugal

**Keywords:** gastrointestinal protectants, acid suppressants, proton pump inhibitors, ACVIM consensus guidelines, gastrointestinal ulceration, steroid-induced gastrointestinal ulceration

## Abstract

In both human and veterinary healthcare, gastrointestinal protectants (GIPs) are considered a staple of clinical practice in that they are prescribed by general practitioners (GPs) and specialists alike. Concerning GIP use, overprescription of proton pump inhibitors (PPIs) has become a growing concern among human healthcare providers. This trend has also been documented within veterinary practice, prompting the American College of Veterinary Internal Medicine (ACVIM) to publish a consensus statement in 2018 concerning evidence-based indications for GIP use. This observational cross-sectional study evaluated self-reported prescribing protocols among Portuguese GPs to determine whether there is adherence to the consensus guidelines. Respondents were Portuguese GPs recruited by social media posts in veterinarian online forums. Data were collected from 124 respondents concerning their GIPs of choice and their rationales for prescribing them. Data were mined for prescription patterns and protocols. Among GIPs, PPIs were prescribed more often. Rationales for use included gastrointestinal ulceration and erosion (GUE), prophylactic management of nonerosive gastritis, pancreatitis, reflux esophagitis, and steroid-induced ulceration. Once-daily administration of PPIs was the most frequent dosing regime among respondents. Ninety-six percent of PPI prescribers advocated that the drug be administered either shortly before or at mealtime. Forty-nine percent of respondents supported long-term use of PPIs. Fifty-nine percent of respondents acknowledged discontinuing PPIs abruptly. This study supports that Portuguese GPs commonly prescribe GIPs in accordance with ACVIM recommendations to medically manage GUE. However, misuse of GIPs does occur, and they have been prescribed where their therapeutic value is debatable. Educational strategies should target GPs in an effort to reduce GIP misuse.

## 1. Introduction

Over the past decade, gastrointestinal protectants (GIPs) have been prescribed by human healthcare providers with increased frequency to manage presumptive dyspepsia [1]. Treatment is often an empirical approach to symptom management rather than evidence based; pharmaceuticals are routinely prescribed without a definitive diagnosis to support GIP use [2]. Proton pump inhibitors (PPIs) are overprescribed and/or misused [3,4,5,6,7]. Although historically, the safety profile of PPIs has not been called into question, recent studies raise concerns that extended use, particularly when unwarranted, may be deleterious. Rebound acid secretion can result from short-term treatment with PPIs, necessitating prolonged use of acid suppressants [8,9].

Physicians’ tendency to overprescribe PPIs has also been documented in veterinary practice [10,11]. A presentation at the 2020 ACVIM Forum disclosed that of 200 randomly selected dogs admitted for a first-time hospitalization in a tertiary referral hospital between a five-year period, only 27% treated with an oral or intravenous PPI had appropriate indication for its use [10]. Out of concern for misuse of GIPs among small-animal general practitioners (GPs), the American College of Veterinary Internal Medicine (ACVIM) published a consensus statement in 2018 outlining the evidence-based practice for appropriate GIP use [12].

Even though the beneficial effects of antacids (e.g., salts of aluminum hydroxide, calcium carbonate, and magnesium hydroxide) are related to their capacity to raise gastric pH, this action is limited on account of insufficient buffering capacity [12]. Antacid formulations containing sodium alginate have proven to more effectively control esophageal reflux in people [13,14] as they are thought to displace and/or neutralize the post-prandial acid pocket [12,13,14]. However, there is no evidence base to support the use of antacids to treat gastrointestinal ulceration and erosion (GUE) or gastroesophageal reflux (GER) disease in dogs and cats [12]. The use of acid-suppressing agents, such as histamine type-2 receptor antagonists (H_2_RAs) or PPIs, is preferred. 

PPIs are weak bases that act through direct inhibition of gastric cell H^+^/K^+^ ATPase pumps [15]. In an acidic environment, the pyridine and benzimidazole nitrogens become protonated. The resultant sulfenamide is the active form of the drug [16]. Sulfenamide binds irreversibly to H^+^/K^+^ pumps to inhibit gastric acid production [16]. This mechanism of action is shared by all of the PPIs (e.g., omeprazole, pantoprazole, lansoprazole, esomeprazole, rabeprazole), although the nature of the pyridine and benzimidazole substituents may produce subtle differences among these agents [16]. Rabeprazole has a faster onset of inhibitory action and a greater effect on intragastric pH after the first dose as compared to omeprazole, which may be explained by its higher pK_a_ [16]. However, PPIs with lower pK_a_, such as pantoprazole, are hypothesized to have more targeted activation in the parietal cells and a higher safety profile, since PPIs with higher pK_a_ are activated over a wider pH range [16,17]. PPIs also bind to different cysteine residues at H^+^/K^+^ pumps. This difference in binding is thought to influence the duration of action [17]. Another difference among PPIs is related to the degree to which they are metabolized by cytochrome P450 2C19 and 3A [17]. This metabolic subtlety yields different degrees of plasma concentrations and elimination half-lives, which influence how human patients respond to PPIs [17]. The biochemical differences between PPIs may help explain the subtle variations in the pharmacological response in human patients, yet there is insufficient evidence that clinical responses in cats and dogs differ when different PPIs are used in the treatment of acid-related disorders [12]. Research supports the use of GIPs to medically manage GUE and prophylactically treat reflux esophagitis because both conditions benefit from acid suppression. PPIs are consistently superior to H_2_RAs when it comes to increasing gastric pH in dogs and cats [18,19,20] Although PPIs do not appear to reduce the number of reflux events [21], they do raise esophageal pH such that reflux is only weakly acidic [21,22]. This alteration in acidity may reduce the severity of reflux esophagitis and its potential to induce esophageal stricture in dogs [12]. The PPI omeprazole has also been proven to be more effective than famotidine, an H_2_RA, at reducing exercise-induced GUE [23]. Therefore, PPIs are considered superior to other GIPs when managing GUE and esophagitis secondary to gastroesophageal reflux (GER) [12]. Appropriate dosing in dogs and cats requires that PPIs be administered every 12 h in order to reach the mean percentage of time of intragastric pH thresholds recommended in humans to heal GUE and erosive esophagitis [18,24].

In addition to the dosing regime, it is critical that PPIs be administered in an appropriate formulation. PPIs are acid labile, meaning that they will be destroyed by gastric pH unless they are prepared in oral formulations that can reach the intestine intact for absorption [25]. Such formulations include capsules with enteric-coated microgranules and enteric-coated tablets [25]. Breaking or crushing either formulation or using a compounded version was once thought to reduce the efficacy of PPIs [12,26]. However, these concerns are unfounded. Fractional doses of omeprazole tablets still provide superior acid suppression in cats as compared to famotidine or placebos [20]. The same holds true for dogs [19]. These findings support the use of fractional dosing of omeprazole in tablet form and/or reformulated paste to make dose adjustments for individual patients [19,20].

PPIs work by blocking proton pumps that have already been activated by the presence of a meal in the stomach [27]. However, a full stomach delays the absorption of PPIs in people, thereby reducing efficacy [28]. This presents a therapeutic challenge for those who prescribe PPIs. Clinicians must determine whether to prescribe PPIs with or without food, yet even the guidelines to which they ascribe lack consistency. According to the ACVIM consensus statement, PPIs should be taken either shortly before (e.g., 30–45 min) or concurrent with a meal [12]. 

What can be agreed upon is that PPIs should not be administered along with H_2_RAs. The latter reduce gastric acid, whereas PPIs rely upon gastric acid to be activated. Because of this, co-administration is contraindicated. H_2_RAs use ultimately impairs the efficacy of PPIs because H_2_RAs inhibit the stimulatory state of acid-producing parietal cells [24,29]. 

Another aspect of dosing that the ACVIM consensus statement addresses is long-term use of PPIs [12]. Human healthcare research has disclosed that long-term users of PPIs may be at increased risk of orthopedic maladies, such as hip or vertebral fractures [30], *Clostridium difficile* infection, and/or recurrence of *Clostridium difficile* infection [31,32,33]. Accordingly, there has been interest in discontinuing unnecessary PPI therapy among human patients. Cessation of therapy is typically handled via a tapered approach because rebound gastric acid hypersecretion (RAH) has been known to occur in those who were prescribed PPIs for extended use [34,35,36].

Despite the frequency with which GIPs are prescribed in veterinary practice, clinicians’ prescribing patterns and the appropriateness of therapy have rarely been characterized. The aim of this study was to evaluate the current GIP prescription trends of small-animal GPs in Portugal to determine whether the ACVIM consensus guidelines are being applied.

## 2. Materials and Methods

An observational cross-sectional study was conducted. A questionnaire was developed to collect information from Portuguese small-animal GPs about their trends in prescribing gastrointestinal protectants. Data were collected between 1 January and 28 February 2020. Participants were recruited for the survey through promotion on online veterinary forums and social media posts. An 18-item electronic survey was used to collect specific data about their indications for prescribing GIPs, their GIPs and PPIs of choice, and their dosing regimens. A copy of the survey will be made available to readers upon request.

Participation in this study was strictly voluntary. No incentive was offered for participation, and all responses were anonymous. Before participating in data collection, each respondent was required to provide informed consent. 

### 2.1. Survey Information

GPs were asked to identify by name the GIPs that they prescribed most often and the indication(s) for use. GPs who acknowledged preferential use of omeprazole were also asked whether to achieve an optimal dosing of commercially available omeprazole formulations, they recommend capsule opening or/and compounded formulations. 

The online questionnaire also asked GPs to consider their use of the PPI omeprazole as compared to other GIPs, such as famotidine. Specifically, respondents were asked whether they had a preference for PPIs over other products and, if so, to justify their preference. To identify the frequency of association therapies with acid suppressants, co-administration of omeprazole with famotidine was also accessed. 

Additional questions asked respondents to disclose their prescribed frequency of administration of omeprazole, whether administration was paired with food, the length of therapy, and withdrawal practices. Furthermore, all participants were asked whether they were aware of and/or had read the ACVIM consensus statement.

### 2.2. Statistical Analysis

All survey questions were multiple choice or checklist design. Data were collected and organized in Microsoft Office Excel^®^. Statistical analysis was performed with R 3.6.3 for Windows. Descriptive statistics were used to analyze the categorical data. Associations between respondents’ preferences and their knowledge of the ACVIM consensus statement were individually accessed using nonparametric tests (either chi-square or Fisher’s exact test). Specifically, we hypothesized that the preference for omeprazole over other products, co-administration of omeprazole with famotidine, the regime of administration, and withdrawal practices is made by GPs regardless of their knowledge of the ACVIM consensus statement. Statistical significance of non-parametric tests was set at <0.05.

## 3. Results

Completed surveys were recorded from 124 respondents throughout Portugal. Data concerning GIP preference are summarized on Table 1. Seventy-seven respondents (62.0%) disclosed that they prescribe PPIs more frequently than any other GIP, including H_2_RAs, antacids, sucralfate, and misoprostol. Sucralfate was preferred by 27 participants (21.7%). H_2_RAs were the most frequent choice of 20 GPs (16.1%), with famotidine being preferred by 18 of them (18/20, 90%). Data are summarized in Table 1. GPs who prescribed acid suppressants more frequently than other GIPs (97/124, 78.2%) mostly preferred omeprazole (71/97, 73.1%). 

Concerning therapeutic indications for use of GIPs, 122 of 124 respondents (98.3%) reported that they prescribe GIPs to medically manage GUE, while 99 of the 124 participants (79.8%) prescribed GIPs for prophylactic management of esophagitis secondary to GER, but only 98 of them (79.0%) used it also to manage GUE. In addition, 122 of 124 GPs (98.3%) prescribed GIPs despite a lack of strong supporting evidence in the veterinary literature to justify their use, such as prophylactic management in dogs and cats with non-erosive gastritis (111/124, 89.5%), prophylactic management of steroid-induced ulceration and erosion (87/124, 70.2%), prophylactic management in dogs and cats with pancreatitis (76/124, 61.3%), prophylactic management in dogs and cats with chronic kidney disease (CKD) (74/124, 59.7%), prevention or management of thrombocytopenia-induced gastrointestinal bleeding (38/124, 30.6%), and prophylactic management in dogs and cats with hepatic disease not associated with gastrointestinal bleeding (27/124, 21.8%). Data are summarized in Table 2. Finally, 54 of 124 GPs (43.5%) were familiar with the ACVIM consensus statement; however, the proportion of participants that prescribed GIPs for evidence-based therapeutic indications was independent of their knowledge of the consensus statement (χ^2^ (1) = 0.557; *p* = 0.455; *n* = 124). 

In the survey, 83 of the 124 respondents (66.9%) preferred omeprazole over other GIPs, and this choice was independent of their knowledge of the ACVIM consensus statement (*p* = 0.565, Fisher’s exact test). The following reasons were provided as a means of justifying this preference: scientific evidence that omeprazole is more effective than other GIPs (45/83, 54.2%), self-reported better experiences involving omeprazole being more efficacious as compared to other GIPs (36/83, 43.4%), the affordability of omeprazole (13/83, 15.6%), and the ease with which commercial formulations of omeprazole can be administered to individual patients (16/83, 19.2%). Between the two most frequently prescribed acid suppressants, 100 of the 124 GPs (80.6%) preferred omeprazole over famotidine and 42 participants (33.9%) reported co-administration of both drugs. Although participants who were familiar with the ACVIM consensus statement administered combination therapies less, this association was not statistically significant (χ^2^ (1) = 0.012; *p* = 0.912; *n* = 124).

Once-daily administration of the omeprazole regime was prescribed by 86 of 124 respondents (69.3%), twice-daily administration was recommended by 30 of 124 GPs (24.1%), while 8 of 124 participants (6.5%) indicated that they adjusted their protocol based on the species, clinical context, and perceived client compliance. The proportion of GPs that recommended once-daily administration of omeprazole was higher than those who had no knowledge of the ACVIM consensus statement (*p* <0.05, Fisher’s exact test). In addition, 106 of 124 respondents (85.5%) prescribed that omeprazole be administered 30 to 45 min before a meal, 13 (13/124; 10.5%) prescribed it with a meal, and 5 (5/124; 4.0%) did not specify the time administration.

The survey also showed that 63 of 124 participants (50.8%) did not recommend long-term omeprazole use, while 61 of 124 (49.1%) prescribed it for a duration that exceeded three weeks. Of those 61, 36 respondents (59.0%) abruptly ceased treatment as soon as justified, while 25 (25/61, 40.9%) tapered the dose to gradually discontinue therapy. There was no statistically significant association between the number of participants who gradually titrated omeprazole and the knowledge of the ACVIM consensus statement (*p* = 0.08, Fisher’s exact test).

**Figure 1:**
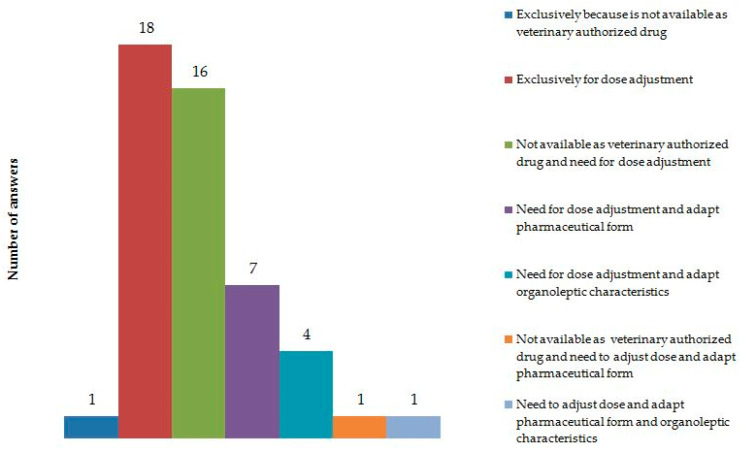
Reasons that justify the use of omeprazole-compounded formulations by Portuguese GPs.

Finally, 49 of 71 GPs who used omeprazole more frequently disclosed their willingness to prescribe compounded formulations. As shown in Figure 1, 18 of 49 GPs (36.7%) justified this treatment because it could more easily undergo dose adjustment to meet the patient’s needs. Forty-seven of forty-nine respondents (95.9%) cited this in addition to one or more reasons: 16 of 49 GPs (32.6%) reported the fact that the active substance is not available as veterinary-authorized medicine in Portugal, 7 (7/49, 1.4%) indicated the need to adapt the pharmaceutical form, 1 (1/49, 2.0%) indicated the need to adapt organoleptic characteristics, and 1 (1/49, 2.0%) reported the fact that the active substance is not available as veterinary-authorized medicine in Portugal and the need to adapt the pharmaceutical form. In addition, 18 of 71 participants (25.3%) who used omeprazole more frequently reported that to achieve optimal dosing of commercially available formulations, they sometimes instructed the patient to open the capsule. Of those who did not subscribe to fractional dosing (53/71, 74.6%), 36 (36/53, 67.9%) acknowledged their use of compounded formulations instead.

## 4. Discussion

This study evaluates GIP prescription trends among Portuguese small-animal GPs to determine whether current recommendations provided by the ACVIM consensus statement are followed. The results disclose that PPIs are the most frequently prescribed GIPs. This may be because PPIs are perceived by GPs to provide prolonged control of both basal and meal-stimulated acid secretion as compared to H_2_RAs and are therefore superior at reducing intragastric acidity [15]. As previously described, famotidine has been found to be superior compared to a placebo and ranitidine but inferior to omeprazole for increasing intragastric pH in healthy dogs and cats [18,20,24]. PPIs also have demonstrated efficacy in healing peptic ulcers and erosions as well as erosive esophagitis [15,37]. PPIs inhibit peptic activity by achieving pH values greater than 4, the critical intragastric pH threshold to stop pepsin activation in humans [15,18]. This is more evident with PPIs rather than H_2_RAs as over a 24-h period, H_2_RAs still allow some proteolytic activity [15]. This may help to explain why famotidine has some beneficial effect in decreasing gastric lesions in racing sled dogs compared with no treatment, although omeprazole shows better results, decreasing the severity and prevalence of these lesions [23]. Despite the evidence that PPIs are more effective for management of acid-related disorders [12], our study reveals that a considerable number (16%) of Portuguese GPs consider H_2_RAs as a preferable choice. We hypothesize that this could be related to the following: (1) H_2_RAs can provide immediate clinical relief, while PPIs can take longer to reach a peak of action [15,17]; (2) PPIs are a more recent drug, while H_2_RAs were historically used among the Portuguese veterinary community; (3) H_2_RAs have additional cytoprotective effects such as increased mucus and bicarbonate secretion [15]; (4) H_2_RAtherapies are well tolerated in dogs and cats, with a good safety profile [38]; and (5) recent studies show potential adverse effects of long-term PPI therapies in cats [39], as well as documented interactions of PPIs with other drugs [40]. Nevertheless, it is important to acknowledge the fact that H_2_RAs are weaker acid suppressants and have proven to be less effective for controlling acid-related problems [15]. Furthermore, even when less potent acid suppression is desired and H_2_RAs are considered a reasonable alternative, it is important to highlight that the continuous administration of these drugs leads to a reduction in acid suppression time [15,41]. This pharmacological tolerance occurs in dogs and cats within 13 days of oral treatment with famotidine [41,42] and can be observed in dogs within 3 days [41].

GIPs are widely prescribed by Portuguese GPs for management of GUE, with 98% of the survey participants using it in these situations. Regardless, only 98 of 124 participants prescribe GIPs for both treating GUE and preventing esophagitis secondary to GER, which corresponds to 79% of respondents who use GIPs in both therapeutic indications supported by the ACVIM consensus statement. Indeed, there is evidence that gastric acid suppression improves healing of GUE and helps prevent erosive esophagitis by increasing the pH of the reflux, with PPIs being superior than other GIPs in both situations and hence being considered a standard for medical treatment of these disorders in dogs and cats [12]. However, 122 of 124 (98%) respondents prescribe GIPs prophylactically when their therapeutic value is questionable. For instance, using GIPs as prophylactic agents in dogs and cats with non-erosive gastritis is considered by 89.5% of participants, even more than the ones who contemplate using it for prophylactic management of reflux esophagitis. In addition, the prescription of GIPs prophylactically in animals with pancreatitis, with CKD, or under therapy with corticosteroids was considered by a significant number of respondents, with 60% or more of them using it in these situations. Most participants were not familiar with the ACVIM consensus statement, which could have helped to explain their choices concerning the therapeutic use of GIPs. However, there was not a statistically significant association between being familiar with the consensus statement and using GIPs for therapeutic indications supported by it, which leads us to think that treatment decisions are based on the individual prescriber’s clinical experience rather than in accordance with an evidence-based approach. Moreover, the theory that the use of GIPs is essential to prevent possible or theoretical drug-related gastric problems or gastrointestinal disorders related to other co-morbidities seems to be established in the veterinary community, even though current scientific evidence does not support it.

The use of omeprazole as a GIP is preferred by most of survey respondents, with 81% favoring this PPI over famotidine. As previously discussed, PPIs have proven to be more effective at raising intragastric pH than famotidine and must be, whenever possible, the preferable choice for management of acid-related gastric problems. There are differences among PPIs, in particular concerning their pharmacokinetic properties, drug interactions, and susceptibility to cytochrome P450 metabolism [16,17]. These differences can translate into slight variations in acid suppression in people [16]. The standard dose of esomeprazole has proven to be more effective compared with standard doses of omeprazole, pantoprazole, lansoprazole, and rabeprazole for controlling gastric acidity, providing an intragastric pH greater than 4 for a greater amount of a 24 h period in human patients [43]. Although the acid suppressant efficacy of intravenous [44,45], oral, and subcutaneous [44] administration of esomeprazole had been proven, with this PPI increasing the mean percentage time in which intragastric pH is ≥3 or ≥4 regardless of the dosing route in healthy dogs, esomeprazole efficacy has not been accessed when compared with other PPIs. Therefore, there is no conclusive evidence that one PPI is more effective for the treatment of acid-induced tissue injury than another in dogs or cats [12].

Prescribing omeprazole and famotidine concurrently was considered by 34% of GPs for treatment of some cases. We hypothesize that this can be related to a believed increase in the acid suppressant effect acquired by the combination of both drugs. However, there is no evidence that co-administration of omeprazole and famotidine is indicated or beneficial for ulcer healing [12], and this combination may even compromise the effectiveness of omeprazole [12,29].

Although the majority of Portuguese GPs recognize the importance of oral administration of PPIs in the short term before (30–45min) or with a meal, the most common dosing regimen among respondents is once-daily administration. This does not meet the current recommendations of the ACVIM consensus statement. In fact, PPIs are most effective when taken in close relation to a meal, with several factors influencing this situation: (1) taking a meal stimulates acid secretion, which, in turn, contributes to the extent of activation of PPIs [27]; (2) intake with a meal may delay peak plasma concentrations, leading to more favorable area under the concentration–time curve of the inhibitory effect of PPIs [23,25]; and (3) a meal buffers intragastric pH, improving PPIs’ intestinal absorption [27]. Dogs, however, do not typically experience the meal buffering effect as human patients do. In fact, a study by Sagawa et al. [46] reported that gastric pH in fed dogs was lower than that in fasted dogs. These conclusions are consistent with the ones of Bersenas et al. [18], who verified that the mean pH and the mean percentage of time the pH was ≥3 or ≥4 were higher under fasting conditions than when dogs were fed. As twice-daily administration of PPIs is not always performed in humans [47,48,49], this can explain why veterinarian GPs choose more often a single-dose administration. However, to approach pH thresholds associated with healing acid-related injuries in people, PPIs should be administered twice daily in dogs and cats [12]. An inadequate regime of administration can lead to an ineffective dose for treatment, thereby raising the concern of improperly conducted therapies, even with appropriate indication for PPI use. Long-term therapies for more than 3–4 weeks with PPIs should be gradually tapered so that RAH can be avoided [12]. Prolonged omeprazole therapy leads to a significant increase in serum gastrin compared with the placebo, which may lead to RAH after abrupt PPI withdrawal [39]. About half of the survey respondents prescribed long-term therapies with omeprazole, and among them, 59% abruptly stopped treatment. This does not meet the current recommendations and can lead to negative effects of the therapy.

The majority of GPs who prescribe omeprazole more frequently report the need to prescribe compounded formulations of the drug in some situations, mostly because the dose of commercially available formulations does not meet the dose requirements for dogs and cats. In fact, available dose formulations of omeprazole in Portugal are 10, 20, and 40 mg, and 1 mg/kg twice daily being the recommended dose for dogs, in a 5 kg dog, GPs can either recommend capsule opening or a compounded formulation. Because the enteric coat that prevents degradation of the drug is on the microgranules, opening the capsule should not have an effect on its pharmacokinetics if microgranules are not crushed or broken. However, to the best of our knowledge, there are no studies concerning this matter; therefore, this practice is not evidence based. The use of compounded formulations of omeprazole has previously been addressed in a restricted number of studies, and the overall conclusion is that its chemical stability depends on the solid dosage form from which compounded formulations are made [50] and the method used to prepare extemporaneous formulations [51]. Twenty-five percent of GPs who use omeprazole more frequently propose opening the capsule to achieve optimal dosing, and among those who do not do it, 68% use compounded formulations to conduct treatment. In addition, other reasons for compounding by participants included the fact that omeprazole is not available as a veterinary-authorized medicine for dogs and cats and the need to adjust organoleptic characteristics and pharmaceutical form of the commercially available formulations. These results illustrate the existing barriers preventing optimal dosing of commercially available omeprazole formulations and the limitations experienced by Portuguese GPs to perform a treatment adapted to dogs and cats.

Because this was a questionnaire-based study, this work had several limitations. Indeed, the fact that it was spread via social networking impacted who chose to respond. Those clinicians who do not make use of social media would have been unlikely to have heard of this study, and therefore, their views remain unheard. Although small-animal GPs represent a major group of veterinarians practicing in Portugal, it was not possible to accurately assess whether the response rate would be higher if the survey was applied to other particular network groups. This is because the online forums in which the survey was spread are not exclusive for small-animal GPs. Nonetheless, these are the most well-known online forums of the country, and the authors believe that the research group is well represented. Specifically concerning the survey, several points such as the use of different GIPs in particular clinical scenarios or the frequency that GPs recommend opening the capsule of omeprazole were not detailed. Although these data would be useful, this would increase the length of the survey, reducing the response rate of the survey. Consequently, only the above-mentioned topics were detailed and discussed.

## 5. Conclusions

Following ACVIM consensus recommendations, GIPs are being prescribed by Portuguese small-animal GPs for GUE and prophylactic management of reflux esophagitis, and PPIs, namely omeprazole, have become their preferred drugs of choice. However, GIPs are still used in situations in which their therapeutic value is questionable, stressing the need for an evidence-based approach to prescribing. Furthermore, the prescribing patterns of omeprazole revealed that PPIs are frequently being administered at incorrect frequencies, in combination with other acid suppressants, and abruptly discontinued in long-term therapies rather than gradually tapered. Occurrence of improperly conducted therapies possibly calls into question the success of the therapy and reinforces the need for GPs’ awareness about GIP use in daily clinical practice.

## Figures and Tables

**Table 1:** Choices of Portuguese small-animal GPs concerning the most frequently prescribed GIPs.

GIPs	GPs Using GIPs (*n*(%)) *n* = 124
Omeprazole	71 (57.2%)
Pantoprazole	5 (4.0%)
Esomeprazole	1(0.8%)
Lansoprazole	0 (0.0%)
Famotidine	18 (14.5%)
Ranitidine	2 (1.6%)
Cimetidine	0 (0.0%)
Sucralfate	27 (21.7%)
Antiacids	0 (0.0%)

**Table 2:** Evidence- and non-evidence-based therapeutic indications for use of GIPs among Portuguese GPs, taking as a reference the ACVIM consensus statement.

Evidence-Based Indications	GPs Using GIPs (*n*(%)) *n* = 124
Treatment of gastrointestinal ulceration or erosion	122 (98.3%)
Prophylactic management of reflux esophagitis	99 (79.8%)
Non-Evidence-Based Indications	
Prophylactic management in animals with non-erosive gastritis	111 (89.5%)
Prophylactic management of steroid-induced ulceration	87 (70.2%)
Prophylactic management in animals with pancreatitis	76 (61.3%)
Prophylactic management in animals with CKD	74 (59.7%)
Prevention or management of thrombocytopenia-induced gastrointestinal bleeding	38 (30.6%)
Prophylactic management in animals with hepatic disease not associated with gastrointestinal bleeding	27 (21.8%)

## Data Availability

As stated, a copy of the survey will be made available to readers upon request.

## References

[1] Haastrup P.F., Rasmussen S., Hansen J.M., Christensen R.D., Søndergaard J., Jarbøl D.E. (2016). General practice variation when initiating long-term prescribing of proton pump inhibitors: A nationwide cohort study. BMC Fam. Pract..

[2] Lassen A., Hallas J., Schaffalitzky De Muckadell O.B. (2004). Use of anti-secretory medication: A population-based cohort study. Aliment. Pharmacol. Ther..

[3] Batuwitage B.T., Kingham J.G.C., Morgan N.E., Bartlett R.L. (2007). Inappropriateprescribingofprotonpumpinhibitorsinprimarycare. Postgrad. Med. J..

[4] Rakesh T.P. (2011). Proton pump inhibitors: Use, misuse and concerns about long-term therapy. Clin. J. Gastroenterol..

[5] Othman F., Card T.R., Crooks C.J. (2016). Proton pump inhibitor prescribing patterns in the UK: A primary care database study. Pharmacoepidemiol. Saf..

[6] Haastrup P., Paulsen M.S., Zwisler J.E., Begtrup L.M., Hansen J.M., Rasmussen S., Jarbøl D.E. (2014). Rapidly increasing prescribing of proton pump inhibitors in primary care despite interventions: A nationwide observational study. Eur. J. Gen. Pract..

[7] Sattayalertyanyong O., Thitilertdecha P., Auesomwang C. (2020). The inappropriate use of proton pump inhibitors during admission and after discharge: A prospective cross-sectional study. Int. J. Clin. Pharm..

[8] Haastrup P.F., Paulsen M.S., Christensen R.D., Søndergaard J., Hansen J.M., Jarbøl D.E. (2016). Medical and non-medical predictors of initiating long-term use of proton pump inhibitors: A nationwide cohort study of first-time users during a 10-year period. Aliment. Pharmacol. Ther..

[9] Lødrup A.B., Reimer C., Bytzer P. (2013). Systematic review: Symptoms of rebound acid hypersecretion following proton pump inhibitor treatment. Scand. J. Gastroenterol..

[10] Duxbury S., Sorah E., Andrews P., Tolbert M.K. Evaluation of proton pump inhibitor use in canine patients hospitalized in a tertiary referral hospital. Proceedings of the ACVIM online Forum.

[11] McCormack R., Olley L., Glanemann B., Swann J.W. (2020). Prospective observational study of the use of omeprazole and maropitant citrate in veterinary specialist care. Sci. Rep..

[12] Marks S.L., Kook P.H., Papich M.G., Tolbert M.K., Willard M.D. (2018). ACVIM consensus statement: Support for rational administration of gastrointestinal protectants to dogs and cats. J. Vet. Intern. Med..

[13] Ruigh A., Roman S., Chen J., Pandolfino J.E., Kahrilas P.J. (2014). Gaviscon Double Action Liquid (antacid & alginate) is more effective than antacid in controlling post-prandial oesophageal acid exposure in GERD patients: A double-blind crossover study. Aliment. Pharmacol. Ther..

[14] Deraman M.A., Hafidz M.I.A., Lawenko R.M., Ma Z.F., Wong M.S., Coyle C., Lee Y.Y. (2020). Randomised clinical trial: The effectiveness of Gaviscon Advance vs non-alginate antacid in suppression of acid pocket and post-prandial reflux in obese individuals after late-night supper. Aliment. Pharmacol. Ther..

[15] Huang J., Hunt R.H. (2001). Pharmacological and pharmacodynamic essentials of H2-receptor antagonists and proton pump inhibitors for the practising physician. Best Pract. Res. Clin. Gastroenterol..

[16] Horn J. (2000). The proton-pump inhibitors: Similarities and differences. Clin. Ther..

[17] Welage L.S. (2003). Pharmacologic properties of proton pump inhibitors. Pharmacotherapy.

[18] Bersenas A.M.E., Mathews K.A., Allen D.G., Conlon P.D. (2005). Effects of ranitidine, famotidine, pantoprazole, and omeprazole on intragastric pH in dogs. Am. J. Vet. Res..

[19] Tolbert K., Bissett S., King A., Davidson G., Papich M., Peters E., Degernes L. (2011). Efficacy of oral famotidine and 2 omeprazole formulations for the control of intragastric pH in dogs. J. Vet. Intern. Med..

[20] Parkinson S., Tolbert K., Messenger K., Odunayo A., Brand M., Davidson G., Peters E., Reed A., Papich M.G. (2015). EEvaluation of the effect of orally administered acid suppressants on intragastric pH in cats. J. Vet. Intern. Med..

[21] Panti A., Bennet R.C., Corletto F., Brearly J., Jeffery N., Mellanby R.J. (2009). The effect of omeprazole on oesophageal pH in dogs during anaesthesia. J. Small Anim. Pract..

[22] Zacuto A.C., Marks S.L., Osborn J., Douthitt K.L., Hollingshead K.L., Hayashi K., Kapatkin A.S., Pypendop B.H., Belafsky P.C. (2012). The influence of esomeprazole and cisapride on gastroesophageal reflux during anesthesia in dogs. J. Vet. Intern. Med..

[23] Williamson K.K., Willard M.D., Payton M.E., Davis M.S. (2010). Efficacy of omeprazole versus high-dose famotidine for prevention of exercise-induced gastritis in racing Alaskan sled dogs. J. Vet. Intern. Med..

[24] Sutalo S., Ruetten M., Hartnack S., Reusch C.E., Hook P.H. (2015). The Effect of Orally Administrated Ranitidineand Once-Daillyor Twice-Daily Orally Administrated Omeprazoleon Intragastricp Hin Cats. J. Vet. Intern. Med..

[25] Sachs G. (1997). Proton pump inhibitors and acid-related diseases. Pharmacotherapy.

[26] Merritt A.M., Sanchez L.C., Burrow J.A., Church M., Ludzia S. (2003). Effect of GastroGard and three compounded oral omeprazole preparations on 24 h intragastric pH in gastrically cannulated mature horses. Equine Vet. J..

[27] Hatlebakk J.G., Katz P.O., Camacho-Lobato L., Castell D.O. (2000). Proton pump inhibitors: Better acid suppression when taken before a meal than without a meal. Aliment. Pharmacol. Ther..

[28] Ochoa D., Román M., Cabaleiro T., Saiz-Rodríguez M., Meija G., Abad-Santos F. (2020). Effect of food on the pharmacokinetics of omeprazole, pantoprazole and rabeprazole. BMC Pharm. Toxicol..

[29] DeGraef J., Woussen-Colle M.C. (1986). Influence of the stimulation state of the parietal cells on the inhibitory effect of omeprazole on gastric acid secretion in dogs. Gastroenterology.

[30] Ngamruengphong S., Leontiadis G., Radhi S., Dentino A., Nugent K. (2011). Proton pump inhibitors and risk of fracture: A systematic review and meta-analysis of observational studies. Am. J. Gastroenterol..

[31] Deshpande A., Pant C., Pasupuleti V., Rolston D.K., Jain A., Deshpande N., Thota P., Sferra T.J., Hernandez A.V. (2012). Association between proton pump inhibitor therapy and Clostridium difficile infection in a meta-analysis. Clin. Gastroenterol. Hepatol..

[32] Janarthanan S., Ditah I., Adler D.G., Ehrinpreis M.N. (2012). Clostridium difficile-associated diarrhea and proton pump inhibitor therapy: A meta-analysis. Am. J. Gastroenterol..

[33] Kwok C.S., Arthur A.K., Anibueze C.I., Singh S., Cavallazzi R., Loke Y.K. (2012). Risk ofClostridium difficileInfection With Acid Suppressing Drugs and Antibiotics: Meta-Analysis. Am. J. Gastroenterol..

[34] Niklasson A., Lindström L., Simrén M., Lindberg G., Björnsson E. (2010). Dyspeptic symptom development after discontinuation of a proton pump inhibitor: A double-blind placebo-controlled trial. Am. J. Gastroenterol..

[35] Inadomi J.M., Jamal R., Murata G.H., Hoffman R.M., Lavezo L.A., Vigil J.M., Swanson K.M., Sonnenberg A. (2001). Step-down management of gastroesophageal reflux disease. Gastroenterology.

[36] Haastrup P., Paulsen M.S., Begtrup L.M., Hansen J., Jarbøl D. (2014). Strategies for discontinuation of proton pump inhibitors: A systematic review. Farm. Pract..

[37] MacFarlane B. (2018). Management of gastroesophageal reflux disease in adults: A pharmacist’s perspective. Integr. Pharm. Res. Pract..

[38] Papich M.G., Reviere J.E., Papich M.G. (2009). Drugs affecting gastrointestinal function. Veterinary Pharmacology & Therapeutics.

[39] Gould E., Clements C., Reed A., Giori L., Steiner J.M., Lidbury J.A., Suchodolski J.S., Brand M., Moyers T., Emery L. (2016). A prospective, placebo-controlled pilot evaluation of the effect of omeprazole on serum calcium, magnesium, cobalamin, gastrin concentrations, and bone in cats. J. Vet. Intern. Med..

[40] Jones S.M., Galer A., Enomoto H., Ishii P., Pilla R., Price J., Suchodolski J., Steiner J.M., Papich M.G., Messenger K. (2020). The effect of combined carprofen and omeprazole administration on gastrointestinal permeability and inflammation in dogs. J. Vet. Inter. Med..

[41] Tolbert M.K., Graham A., Odunayo A., Price J., Steiner J.M., Newkirk K., Hecht S. (2017). Repeated famotidine administration results in a diminished effect on intragastric pH in dogs. J. Vet. Intern. Med..

[42] Golly E., Odunayo A., Daves M., Vose J., Price J., Hecht S., Steiner J.M., Hillsman S., Tolbert M.K. (2019). The frequency of oral famotidine administration influences its effect on gastric pH in cats over time. J.Vet.Intern.Med..

[43] Miner P., Katz P.O., Chen Y., Sostek M. (2003). Gastric acid control with esomeprazole, lansoprazole, omeprazole, pantoprazole, and rabeprazole: A five-way crossover study. Am. J. Gastroenterol..

[44] Hwang J.-H., Jeong J.-W., Song G.-H., Koo T.-S., Seo K.-W. (2017). Pharmacokinetics and acid suppressant efficacy of esomeprazole after intravenous, oral, and subcutaneous administration to healthy beagle dogs. J. Vet. Inter. Med..

[45] Seo D.-H., Lee J.-B., Hwang J.-H., Jeong J.-W., Song G.H., Koo T.-S. (2019). Pharmacokinetics and pharmacodynamics of intravenous esomeprazole at 2 different dosages in dogs. J. Vet. Inter. Med..

[46] Sagawa K., Fasheng L., Ryan L., Sutton S.C. (2009). Fed and fasted gastric pH and gastric residence time in conscious beagle dogs. J. Pharm. Sci..

[47] Khalaf M., Abdul-Hussein M., Castell D. (2016). Are Twice Daily Proton Pump Inhibitors Better Than Once Daily in Suspected GERD? 429?. Am. J. Gastroenterol.

[48] Zhang H., Yang Z., Ni Z., Yongquan S. (2017). A meta-analysis and systematic review of the efficacy of twice daily PPIs versus once daily for treatment of gastroesophageal reflux disease. Gastroenterol. Res. Pract..

[49] Ayoub F., Khullar V., Banerjee D., Stoner P., Lambrou T., Westerveld D.R., Hanayneh W., Kamel A.Y., Estores D. (2018). Once Versus Twice-Daily Oral Proton Pump Inhibitor Therapy for Prevention of Peptic Ulcer Rebleeding: A Propensity Score-Matched Analysis. Gastroentrol. Res..

[50] Meissner S., Bansal M., Dela C.P., Hanning S., Svirskis D. (2020). The Effect of Manufacturer on the Compounding of Omeprazole Suspensions and Their Stability Assessment. Int. J. Pharm. Compd..

[51] Garg S., Svirkis D., Al-Kabban M., Farhan S., Komeshi M., Lee J., Liu Q., Naidoo S. (2009). Chemical stability of extemporaneously compounded omeprazole formulations: A comparison of two methods of compounding. Int. J. Pharm. Compd..

